# Interferon-β Pretreatment of Conventional and Plasmacytoid Human Dendritic Cells Enhances Their Activation by Influenza Virus

**DOI:** 10.1371/journal.ppat.1000193

**Published:** 2008-10-31

**Authors:** Hannah Phipps-Yonas, Jeremy Seto, Stuart C. Sealfon, Thomas M. Moran, Ana Fernandez-Sesma

**Affiliations:** 1 Department of Microbiology, Mount Sinai School of Medicine, New York, New York, United States of America; 2 Center for Investigating Viral Immunity and Antagonism, Department of Microbiology, Mount Sinai School of Medicine, New York, New York, United States of America; 3 Department of Neurology, Mount Sinai School of Medicine, New York, New York, United States of America; Washington University in Saint Louis School of Medicine, United States of America

## Abstract

Influenza virus produces a protein, NS1, that inhibits infected cells from releasing type I interferon (IFN) and blocks maturation of conventional dendritic cells (DCs). As a result, influenza virus is a poor activator of both mouse and human DCs in vitro. However, in vivo a strong immune response to virus infection is generated in both species, suggesting that other factors may contribute to the maturation of DCs in vivo. It is likely that the environment in which a DC encounters a virus would contain multiple pro-inflammatory molecules, including type I IFN. Type I IFN is a critical component of the viral immune response that initiates an antiviral state in cells, primarily by triggering a broad transcriptional program that interferes with the ability of virus to establish infection in the cell. In this study, we have examined the activation profiles of both conventional and plasmacytoid dendritic cells (cDCs and pDCs) in response to an influenza virus infection in the context of a type I IFN-containing environment. We found that both cDCs and pDCs demonstrate a greater activation response to influenza virus when pre-exposed to IFN-β (IFN priming); although, the priming kinetics are different in these two cell types. This strongly suggests that type I IFN functions not only to reduce viral replication in these immune cells, but also to promote greater DC activation during influenza virus infections.

## Author Summary

Influenza infection leads to a serious respiratory infection of the lung epithelium. Lying directly below the epithelial cells are immune system sentinels known as dendritic cells. These cells interact with the virus and carry parts of the virus to draining lymph nodes to activate killer T cells. In order to effectively carry out this function, DCs must perceive the presence of a virus using receptors specially adapted for this function. However, when DCs are mixed with influenza virus in the laboratory, no activation occurs because the virus produces a protein called NS1 that blocks the receptors. Yet, patients infected with influenza virus develop a strong adaptive response that leads to recovery from infection. This observation suggests that additional factors must be present that contribute to the activation of the DCs. The most likely contributor is type I interferon, a ubiquitous protein released from many cells upon exposure to virus. In this study, we mixed influenza virus with DCs in the presence of type I interferon and found that this greatly enhanced their activation. Treatment with interferon allowed the DC to bypass the block in activation mediated by the influenza NS1 protein. Our data suggest that the production of type I interferon within an infected patient may endow the DCs with the ability to fully respond to influenza virus.

## Introduction

Dendritic cells (DCs) play a key role in the initiation and regulation of the immune system. They respond to various microbial stimuli by undergoing a process of activation that propels them to migrate to draining lymph nodes and endows them with the ability to efficiently activate T cells [1],[2]. The process of DC activation involves several steps including upregulation of surface markers, cytokine and chemokine secretion and the ability to leave the tissue and migrate to draining lymph nodes, and is also known as DC maturation. Depending on the nature of the stimulus maturation is signified by the up-regulation of MHC and co-stimulatory molecules, as well as the secretion of some mixture of cytokines and chemokines that may include type I interferons (IFN-α and IFN-β), IL-6, IL-12, TNF-α, IL-8, IP-10, RANTES and MIP-1β [2],[3].

In response to a viral infection, DCs can be activated by two separate pathways: a toll like receptor (TLR)-dependent and a TLR-independent pathway. The TLR-dependent pathway is made up of several different TLRs that bind specific pathogen-associated-molecular-patterns (PAMPs). TLR 3, 7/8 and 9 are the sensors for viral PAMPs recognizing double-stranded RNA (dsRNA), single-stranded RNA (ssRNA) and CpG DNA motifs, respectively [4]. These TLRs are localized to the endosome and signal via adaptor proteins to induce DC activation [5]. The TLR-independent or internal pathway primarily consists of retinoic acid-inducible gene-I (RIG-I) protein and melanoma differentiation-associated gene product (MDA-5) both located in the cytoplasm (RIG-I like receptors or RLR). RIG-I recognizes cytoplasmic uncapped 5′- tri-phosphate RNAs and MDA-5 recognizes cytoplasmic dsRNA [6].

Conventional DCs (cDCs) are considered the prototypic DCs as they are proficient at presenting antigens and activating T cells [2]. The internal pathway has been shown to play a more significant role in the activation of cDCs to RNA viruses than the TLR-dependent pathway [7],[8]. Plasmacytoid DCs are a second subset of circulating human DCs, that in contrast to cDCs, use the TLR-dependent pathways, specifically TLR7 and TLR9, for activation in response to viruses [7],[9].

Type I IFN is a critical component of the viral immune response. Its expression is highly regulated and pDCs serve as the primary producers of type I IFN in the body [10]. However, virtually all nucleated cells are capable of producing IFN and possess the IFN receptor, endowing them with the ability to respond to type I IFN [11],[12]. Type I IFN initiates an antiviral state by stimulating the transcription of over 200 IFN-responsive genes, some of which code for proteins that interfere with the ability of viruses to establish infection in the cell [13]. Important IFN response genes include MxA, IP-10, ISG54, RIG-I and PKR, among others [13],[14]. Demonstrating the in vivo importance of the type I IFN response is the observation that most successful viruses contain IFN antagonists which act to suppress the IFN pathway either at the level of IFN expression, IFN signaling or the antiviral effects of IFN-responsive proteins [15].

Influenza virus contains a potent IFN antagonist, the NS1 protein, which efficiently blocks type I IFN release from infected cells, including cDCs [16],[17],[18],[19],[20]. Moreover, the NS1 of influenza virus has been shown to block virus triggered activation of cDCs in vitro resulting in poor T cell stimulation [16],[17]. These observations are in contrast to those observed in vivo where fully mature cDCs can be identified in the draining lymph nodes of infected mice and a potent and protective immune response is generated [21]. Thus, in vivo other factors are contributing to the maturation of influenza infected DCs [22],[23]. The most likely factor contributing to the enhancement of DC maturation in vivo is type I IFN [23],[24].

Supporting this hypothesis, Pollara *et. al.* demonstrated type I IFN can prime cDCs to overcome a viral blockade produced during Herpes Simplex Virus (HSV) infections and Osterlund *et. al.* reported that pre-treating cDCs with IFN-α enhanced influenza A virus induced expression of TNF-α, IFN-α, IFN-β and IL-29 genes [23],[25]. Furthermore, mouse DCs have been shown to require type I IFN signaling in order to fully mature following infection with Newcastle disease virus (NDV) and murine cytomegalovirus (MCMV) [26],[27],[28] . Thus in addition to its antiviral effects, type I IFN may also function as an enhancer of DC maturation and may explain the discrepancy observed between the in vitro and in vivo response of cDCs to influenza virus infection. In this study, we systematically examined the influence of type I IFN on the activation profile of cDCs and pDCs in response to an influenza virus infection. We found that cDCs demonstrate a greater activation response to influenza virus when pre-exposed to IFN-β (IFN priming). Additionally, pretreatment of pDCs with IFN augments their ability to release cytokines although the priming kinetics of the two DC types differs significantly. This strongly suggests that type I IFN functions not only to reduce viral replication in cells but promote greater DC activation during influenza virus infections.

## Results

### Impact of IFN dose and pre-exposure time on virus replication

**Figure 1:**
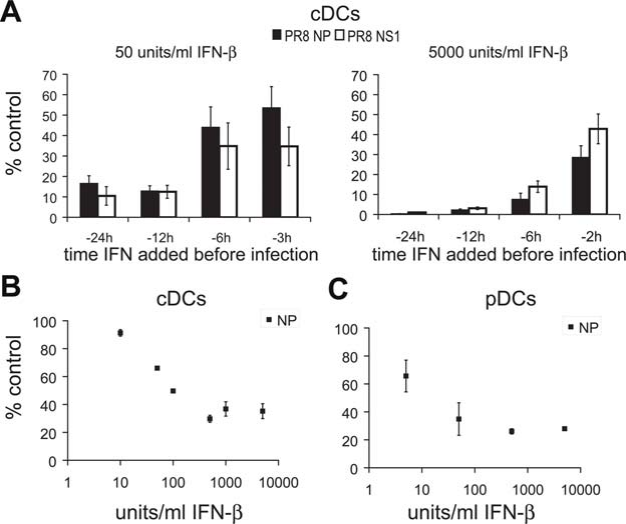
Impact of IFN dose and pre-exposure time on virus replication. (A) cDCs were incubated with IFN-β (50 or 5,000 units/ml) for time indicated. Following pretreatment, the media was removed and fresh media was added along with PR8 virus for a 12-hour infection. (B) cDCs were pretreated for 3 hours with between 5 and 5,000 units/ml IFN-β before a 12-hour infection with fresh media. (C) pDCs were pretreated for 3 hours with between 5 and 5,000 units/ml IFN-β before 8-hour infection with fresh media. (A–C) All results are depicted as percent control, which is the ratio of mRNA copy number of the influenza virus gene NP from samples infected with virus pretreated with IFN-β compared to cells without pretreatment. Mean of samples is depicted with error bars representing the standard deviation of each sample. Data are representative of at least three independent experiments.

Type I IFN initiates an antiviral state in cells and inhibits viral replication [15],[29]. However, viruses differ in their sensitivity to the antiviral effects of IFN [30]. To examine the effects of type I IFN on the ability of human DCs to be infected by influenza virus, we performed a dose and time titration of IFN-β exposure in GM-CSF+IL-4 monocyte-derived DCs (hereafter referred to as ‘cDCs’). Figure 1 shows the impact of treatment with IFN-β on the replication of influenza virus as measured by qRT-PCR of influenza PR8 (PR8) viral product, NP protein. The results are expressed as the percent of the copy number for the NP gene relative to cells infected without IFN treatment. The cells were pretreated for 2, 3, 6, 12, or 24 hours with the indicated amount of IFN, after which the IFN was removed and the cells infected with virus. Virus replication was measured by qRT-PCR at 12 hours post infection (p.i.). Only pretreatment for 24 hours with the highest dose (5,000 units/ml) of IFN-β was able to completely prevent virus replication in DCs. Using the lower dose of IFN-β (50 units/ml) the impact on virus replication was relatively minor when the pre-incubation time was less than 6 hours for both cDC and pDC (Figure 1A and data not shown). Regardless of the length of pretreatment, the low dose of IFN was unable to completely inhibit virus replication. Figure 1B and 1C show the relative sensitivity of cDCs and pDCs to a three hour pretreatment with the indicated concentrations of IFN-β. The results demonstrate that IFN pretreatment reduces the ability of influenza virus to replicate but eliminates replication only with a high concentration and long incubation time.

### Kinetics of gene transcription following a three hour pulse with IFN-β

**Figure 2:**
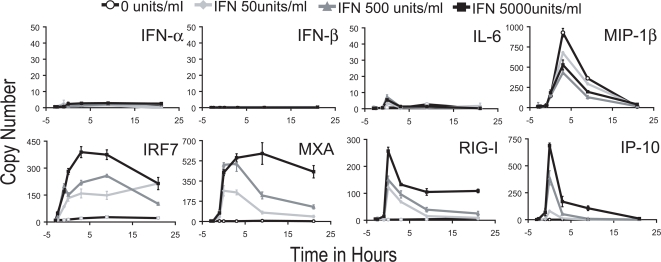
Kinetics of gene transcription following a three hour IFN-β pulse. After 3 hours pretreatment of cDCs with IFN-β (0, 50, 500, or 5,000 units/ml), IFN media was removed and fresh media was added. mRNA expression profiles were determined at 0.5, 1, 3, 6, 12, and 24 hours after IFN addition. IFN removal occurred at 0 hour. Standard deviation of each sample is depicted with data representative of at least two independent experiments.

After IFN-β treatment of human DCs we observed that genes coding for antiviral proteins such as MxA, viral sensors such as RIG-I, transcription factors like STAT1 and IRF7, and chemokines like IP-10 are upregulated (Figure 2). In these experiments cells were pretreated with the indicated concentration of IFN-β for 3 hours after which the cytokine was removed. MxA, STAT1 and IRF7 remain activated for a prolonged period after IFN is removed but mRNA for RIG-I and particularly IP-10 are quickly extinguished when the cytokine is withdrawn. In contrast, most of the other genes associated with DC maturation were not significantly upregulated by IFN treatment including IFN-α, IFN-β, IL-6, and MIP-1β (which was inhibited by IFN treatment). Gene activation was monitored at the indicated time points over a 24 hour period. Thus, IFN-β pretreatment does not result in global gene profile changes in DCs, but rather affects select genes with varying activation kinetics.

### Pretreatment with IFN-β primes DCs to respond more efficiently to virus infection

**Figure 3:**
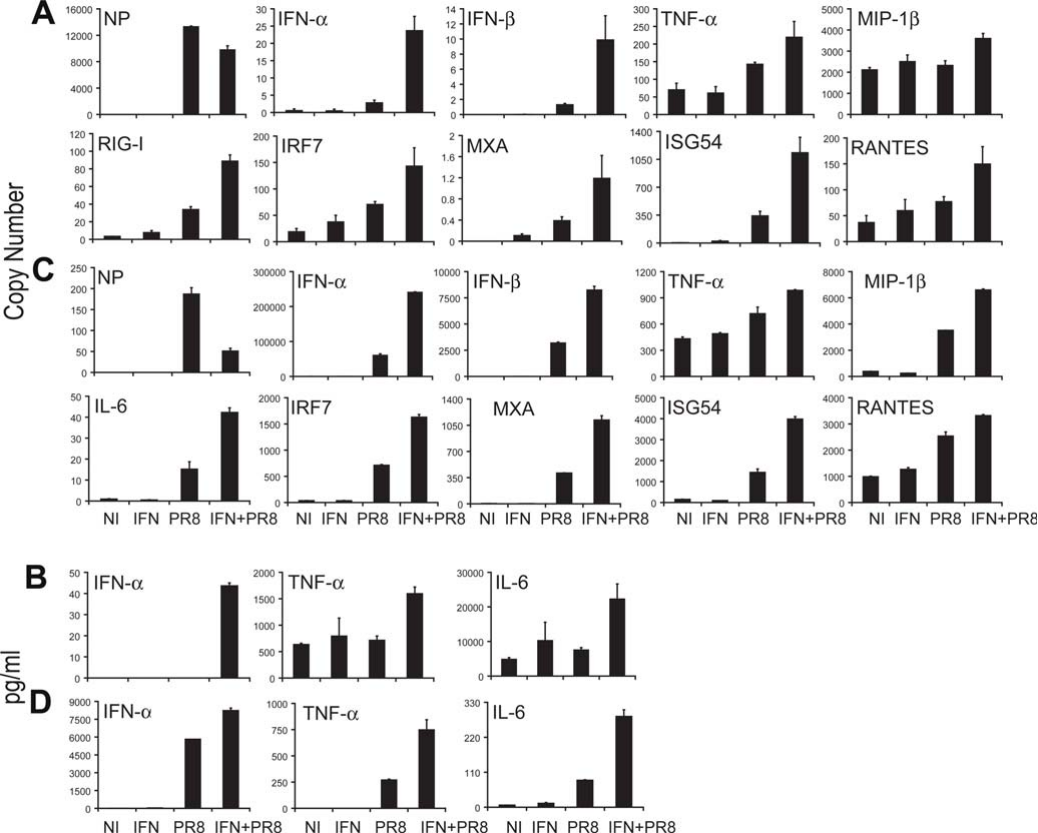
Pretreatment with IFN-β primes DCs to respond more efficiently to virus infection. (A,B) cDCs were pretreated with IFN-β (50 units/ml) for 3 hours. Following pretreatment, the IFN media was removed and cells were infected with PR8 virus (IFN+PR8) for 12 hours. Experiment was done in triplicate with error bars representing standard deviation between samples. All graphs have student t test p<0.05 between the IFN+PR8 condition and other conditions, with the exception of MIP-1β with p>0.05. (C,D) pDCs were pretreated with IFN-β (50 units/ml) for 3 hours. Following pretreatment, the IFN media was removed and cells were infected with PR8 virus (IFN+PR8) for 12 hours. Control pDCs were either infected only (PR8), pretreated with IFN only (IFN), or neither (NI). Mean of samples is depicted with error bars representing the standard deviation of each sample. All graphs have student t test p<0.05 between the IFN+PR8 condition and other conditions. (A,C) Copy number of mRNA expression values are depicted for the specific gene labeled. (B,D) Protein secretion amounts from multiplex ELISAs. Data are representative of at least 5 independent experiments.

cDCs infected with PR8 virus demonstrate a minimal activation profile when compared to the profile observed after infection with viruses such as NDV or Sendai virus [17],[31]. However, cDCs that have been pretreated with a low dose of IFN-β for 3 hours prior to PR8 virus infection demonstrate a substantial increase in mRNA expression for numerous DC activation genes (Figure 3A). Viral RNA expression was moderately decreased in IFN pretreated samples, while all IFN-responsive genes tested demonstrated substantial increases above the level from IFN-β alone following infection with PR8 virus. Moreover, genes not activated by IFN showed enhanced activation following the three hour pretreatment of IFN-β and PR8 virus infection (Figure 3A). This priming effect was not limited to transcription since protein release was equivalently increased (Figure 3B).

In contrast to cDCs, pDCs are highly activated by PR8 virus infection (Figure 3C). Despite the increased basal level of pDC activation following exposure to PR8 virus, pDCs were further primed by IFN-β pretreatment to produce higher levels of mRNA and secrete more protein (Figure 3C and 3D). In conclusion, prior exposure to IFN-β promotes stronger DC activation in both cDCs and pDCs after infection by PR8 virus.

### Impact of interferon treatment on cDC and pDC following virus exposure

**Figure 4 ppat-1000193-g004:**
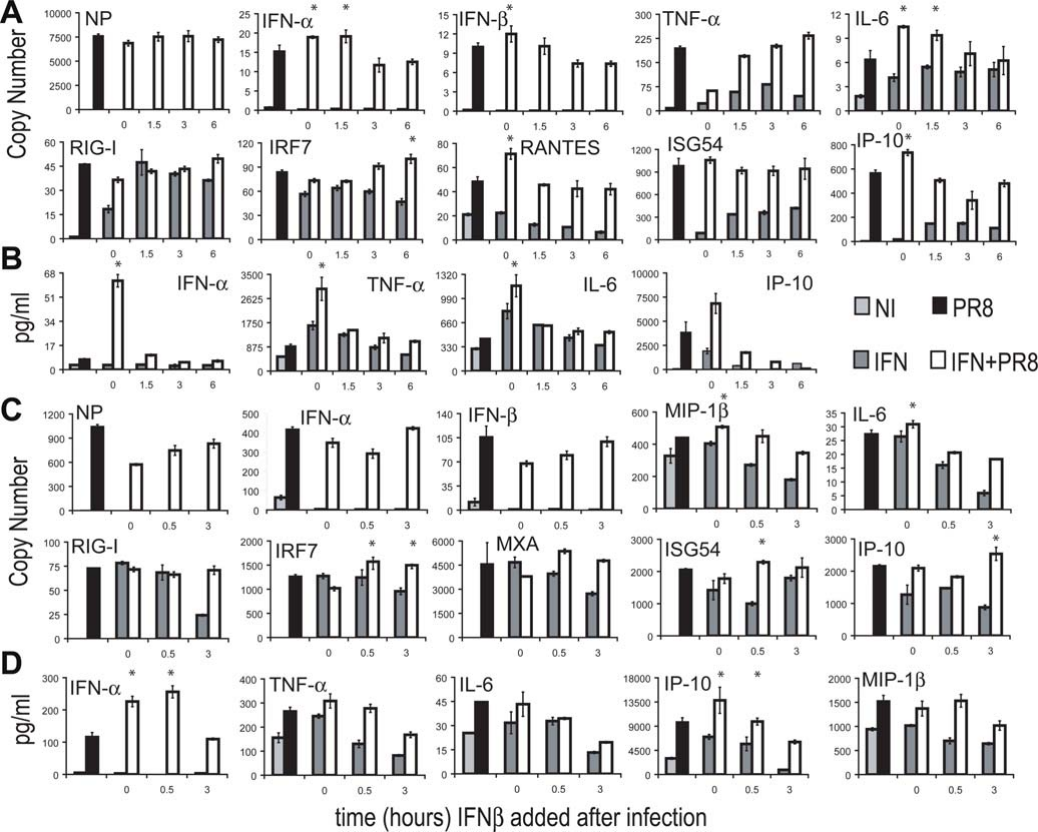
Effect of IFN treatment on cDC and pDC following virus exposure. (A,B) cDCs were infected with PR8 virus treated with IFN-β (50 units/ml) at 0, 1.5, 3, and 6 hours p.i. After 8-hour infection, the supernatants were collected and RNA was isolated. (C,D) pDCs were infected with PR8 virus treated with IFN-β (50 units/ml) at 0, 0.5, and 3 hours p.i. After 4-hour infection, the supernatants were collected and RNA was isolated. Control DCs have only PR8 virus (PR8) or IFN added (IFN) or neither (NI). (A,C) Copy number of mRNA expression values are depicted for the specific gene labeled. (B,D) Protein secretion amounts from multiplex ELISAs. Mean of samples are depicted with error bars of the standard deviation of each sample. Data are representative of at least three independent experiments. Samples with student t test p<0.05 between the IFN+PR8 condition, and the other conditions are marked with *.

IFN-β pretreatment led to enhanced transcription and release of proteins from both subpopulations of DCs following virus infection. In order to determine whether exposure to IFN after virus infection would have a similar effect, cDCs were infected with PR8 virus at the 0 hour, and IFN-β (50 units/ml) was added at 0, 1.5, 3 and 6 hours post infection and left in the culture medium until the mRNA expression profile of the treated cDCs was analyzed 8 hours post infection. Specific viral RNA expression was inhibited by IFN-β as shown in Figure 4 with the highest inhibition of viral NP gene expression observed when IFN-β was added at the same time as the virus. Despite this reduction in viral replication, cDC priming for many genes was most enhanced at 0 hours post infection (Figure 4A) and decreased to basal levels from that point on. This priming effect was observed for both IFN-α and IFN-β genes, as well as genes IFN-responsive and IFN-independent (Figure 4A). Consistent with the mRNA expression patterns, similar results were observed at the protein level (Figure 4B).

When pDC were tested for priming by type I IFN after viral infection, we observed a similar trend but smaller magnitude to that seen with cDCs. Priming was minimally seen only at the early time points for IFN-α and IP-10 and the effect diminished when interferon was added at later time points (Figure 4C and 4D). These data argue that the enhancing effect of IFN-β on DC activation occurs also if it is given immediately after virus infection, but decreases as time after infection increases.

### Optimal DC priming is dependent on the time of IFN-β exposure

**Figure 5 ppat-1000193-g005:**
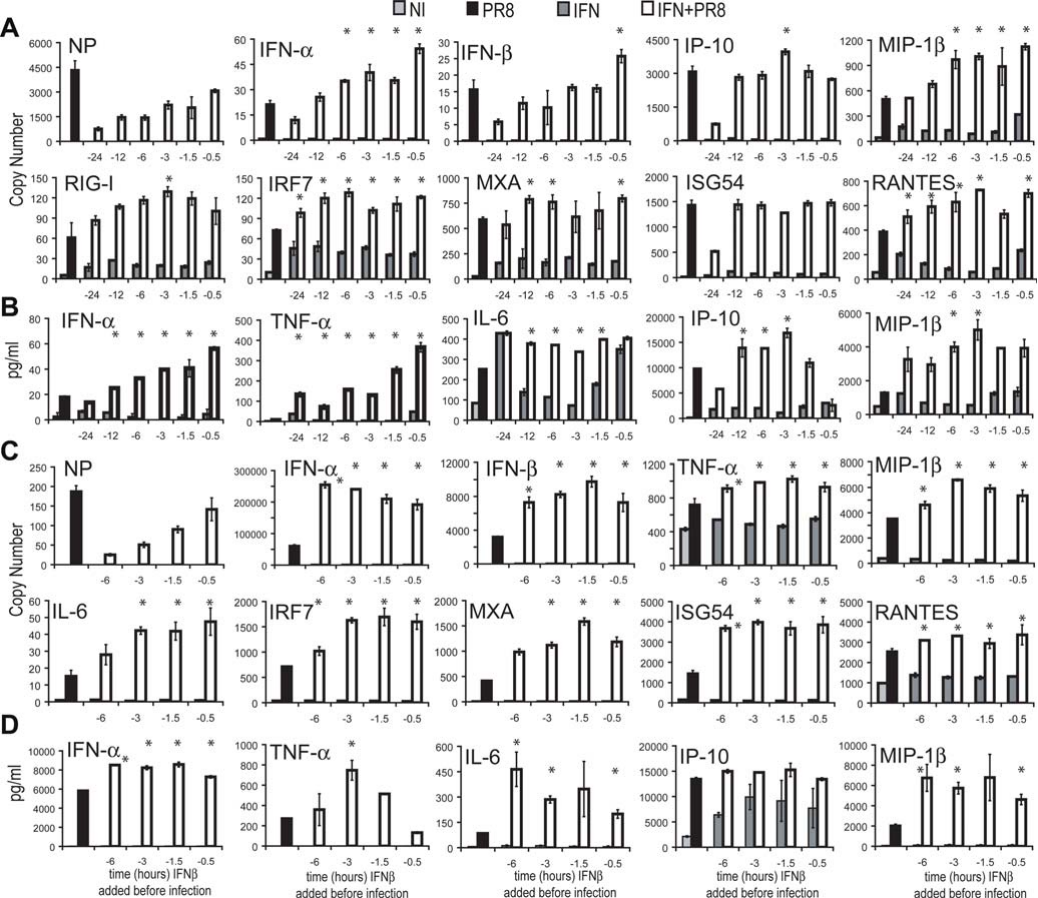
Kinetics of IFN-β priming. (A,B) cDCs were pretreated with IFN-β (50 units/ml) for 24, 12, 6, 3, 1, and 0.5 hours prior to 12-hour infection with PR8 virus in fresh media. (C,D) pDCs were pretreated with IFN-β (50 units/ml) for 24, 12, 6, 3, 1, and 0.5 hours prior to 4-hour infection with PR8 virus in fresh media. Control DCs have only PR8 virus infection (PR8) or IFN pretreatment (IFN) or neither (NI). (A,C) Copy number of mRNA expression values are depicted for the specific gene labeled. (B,D) Protein secretion amounts from multiplex ELISAs. Mean of samples are depicted with error bars of the standard deviation of each sample. Data are representative of at least three independent experiments. Samples with student t test p<0.05 between the IFN+PR8 condition, and the other conditions are marked with *.

In order to determine the optimal time of IFN-β pretreatment needed to maximize DC activation, cDCs were pretreated with IFN-β (50 units/ml) for several intervals between 24 and 0.5 hours, prior to a 12 hour PR8 virus infection. Pretreatment with IFN-β for 1.5–6 hours led to optimal expression of DC activation genes and proteins in cDCs (Figure 5A and 5B). Priming occurred for IFN-α, IFN-β, IFN-responsive genes, and IFN-independent genes (Figure 5A). Surprisingly, cDCs incubated in IFN-β for 12 hours or longer became less responsive to the priming effect as compared with the shorter time points (Figure 5A). This may somewhat reflect the reduced replication of the virus after the prolonged pretreatment.

Due to cell number limitations and cell viability issues; the time course of pDC exposure to IFN-β was shortened (Figure 5C and 5D). Similar to the results seen in cDCs, virus replication was inhibited best in cells exposed to IFN for the longest interval. As a result of the shorter kinetics utilized with pDCs it is difficult to ascertain precisely the optimum pretreatment time, however, it is clear that pretreatment with IFN enhances the response of pDCs to influenza virus infection over a broad time range. Protein secretion from both cell types confirms the priming effects observed in RNA expression in cDC and pDCs (Figure 5B and 5D).

### IFN-β priming of DCs occurs throughout the course of infection with different kinetics in cDCs and pDCs

**Figure 6:**
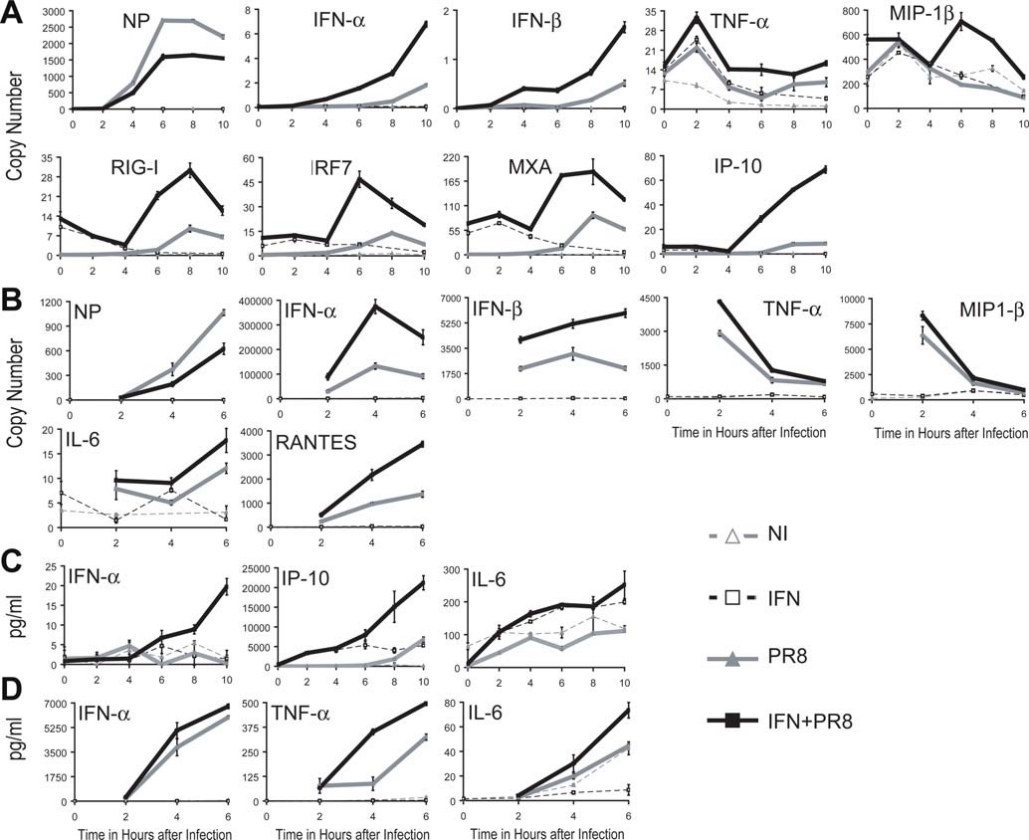
Kinetics of gene activation following IFN-β priming in cDCs and pDCs. (A,B) cDCs were pretreated with IFN-β (50 units/ml) 3 hours prior to infection with PR8 virus in fresh media. Infection was stopped at 0, 2, 4, 6, 8, and 10 hours p.i. Control cDCs have only PR8 virus infection (PR8) or IFN pretreatment (IFN) or neither (NI). (C,D) pDCs were pretreated with IFN-β (50 units/ml) 3 hours prior to infection with PR8 virus in fresh media. Infection was stopped at 0, 2, 4, and 6 hours p.i. Control pDCs have either PR8 virus infection (PR8) or IFN pretreatment (IFN) or neither (NI). (A,C) Copy number of mRNA expression values are depicted for the specific gene labeled. (B,D) Protein secretion amounts from multiplex ELISAs. Mean of samples are depicted with error bars of the standard deviation of each sample. Data are representative of at least three independent experiments.

DCs do not get productively infected with influenza virus though the virus causes an abortive infection with viral message synthesis peaking at between 6–8 hours [32]. To determine the time points where the synergy between the viral and IFN triggering activity is maximal, a time course of infection was performed. cDCs, following a 3 hour pretreatment of IFN-β (50 units/ml), were infected with PR8 virus and samples were collected and analyzed for gene transcription and protein secretion at time points beginning at 0 hours and ending at 10 hours (Figure 6A). Viral mRNA levels show that the peak of viral replication occurs between 6 and 8 hours but were reduced in the IFN treated cells at all time points. Early stimulation of transcription can be observed for the IFN responsive genes but they are not enhanced by simultaneous infection at early time points. However beginning at 4–6 hours after infection the synergistic effect of IFN and infection is seen and correlates with maximal viral gene transcription (Figure 6A).

Due to pDC cell number limitations, the time course of infection for pDCs was shortened (Figure 6B and 6D). Similar to the RNA expression trends in cDCs, viral replication was reduced at all time points assayed in IFN+PR8 samples as compared to PR8 samples. In contrast to cDCs, the synergistic effect of virus and IFN treatment was observed earlier with pDC than with cDCs. This was true with both IFN-responsive and IFN-independent genes. The different kinetics observed with concomitant IFN treatment and infection most likely reflects different activation mechanisms used by the DC subtypes. pDC can be activated through a TLRs mechanism independent of virus replication, while cDCs signal by the virus replication dependent RLR pathway.

Both DCs subsets, show similar results at the level of protein secretion (Figure 6C and 6D). Regardless of the protein type, all cytokines and chemokines tested demonstrated an increase in secretion levels with time (Figure 6C and 6D).

### pDC but not cDCs exposed to a low dose of IFN-β prior to TLR ligand activation demonstrate DC activation priming

To determine if the IFN-β priming was unique to live virus responses, the robustness of priming was compared between live influenza virus and several TLR ligands; in cDCs UV-inactivated virus, poly (I∶C) (TLR 3 ligand), CL-075 (TLR 7/8 ligand), and LPS (TLR 4 ligand) were used and in pDCs Gardiquimod (TLR 7 ligand), CpG, (TLR 9 ligand) and UV-inactivated PR8 virus were used. The DCs were pretreated for 3 hours with the low dose of IFN-β (50 units/ml) and treated with TLR ligands for between 0–12 hours. For each ligand, both dose and time courses were performed and the time point with the greatest priming (the largest differences between samples treated with IFN-β and TLR ligand compared to the other conditions) was determined. Table 1 represents the robustness of priming, as determined by the fold increase of mRNA expression of IFN+TLR ligand over the amount of expression from the IFN alone sample and TLR alone samples [IFN+TLR ligand sample / (IFN alone sample+TLR alone sample)] (Table 1, top part). Contrary to the significant IFN-β priming observed when cDCs were infected with live virus, only small differences were seen in cDCs mRNA expression or protein secretion levels (data not shown) regardless of exposure to IFN-β prior to TLR ligand addition (Table 1, top part). This demonstrates that IFN-β priming in cDCs may be unique to live virus and or activation by RLRs.

**Table 1:** pDCs but not cDCs exposed to a low dose of IFN-β prior to TLR ligand activation demonstrate DC activation priming.

cDCs	IFN-α	IFN-β	TNF-α	IL-6	IP-10	MIP-1β	RANTES	RIG-I	MXA
**PR8**	28.45	13.61	7.13	8.64	37.72	9.66	n/d	7.14	12.88
**PR8-UV**	0.00	0.00	0.64	0.59	2.16	0.44	0.75	0.92	1.97
**poly I∶C**	0.90	2.12	1.08	1.20	1.58	0.73	0.56	0.63	0.85
**CL-075**	0.00	0.00	3.01	4.06	0.17	0.80	3.28	1.42	1.82
**LPS**	0.53	1.75	2.10	0.51	0.84	0.91	0.69	0.37	0.56

pDCs	IFN-α	IFN-β	TNF-α	IL-6	IP-10	MIP-1β	RANTES	IRF7	MXA
**PR8**	5.18	3.38	1.22	1.73	1.32	4.45	3.32	0.65	0.94
**PR8-UV**	1.44	1.57	1.07	0.00	0.60	1.09	1.57	0.87	0.21
**CpG**	1.69	1.31	1.15	2.20	0.92	0.71	0.99	0.40	0.70
**Gard.**	4.91	2.62	1.78	1.73	0.06	0.87	2.25	1.55	1.17

cDCs and pDCs were pretreated with IFN-β (50 units/ml) 3 hours prior to treatment with live influenza PR8 virus, or UV-inactivated influenza, or LPS, or CpG, or poly (I∶C), or CL-075 Gardiquimod (Gard). The robustness of priming is given as the fold increase of mRNA expression of IFN+TLR ligand over the amount of expression from the IFN alone sample and TLR alone samples [IFN+TLR ligand sample / (IFN alone sample+TLR alone sample)]. Data are representative of at least two independent experiments in which both dose and time courses of activation were done. n/d signifies not determined.

In contrast to the cDCs, pDCs demonstrated significant priming with the TLR 7 ligand, Gardiquimod (Gard) for most genes examined and to a lesser degree with UV-inactivated virus and CpG DNA (Table 1, bottom part, and Figure S1). The priming of pDCs with TLR 7 ligand is consistent with TLR 7 being the primary influenza viral sensor in the cell [7],[9]. This increase in mRNA expression was consistent with protein secretion levels (Figure S1 and data not shown).

Overall, these data suggest that there are differences between type I IFN priming in the DC subsets that follow with their pathways of viral activation. In cDCs, activation by TLR agonist is not significantly enhanced by IFN pretreatment, while in pDCs, substantial enhancement is seen with the appropriate TLR ligand.

### IFN priming allows cDCs to partially overcome inhibition by IFN antagonist protein of the influenza virus

**Figure 7:**
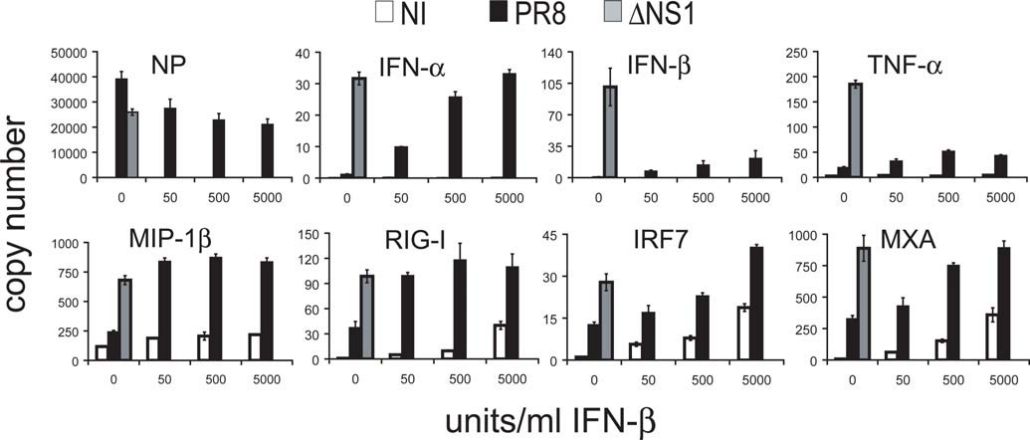
IFN priming allows cDCs to partially overcome inhibition by IFN antagonist protein of the influenza virus. cDCs were either infected with PR8 virus (PR8) or PR8 virus lacking the NS1 protein (ΔNS1) or left non-infected (NI). NI and PR8 samples were also pretreated with IFN-β (50, 500, 5,000 units/ml) for 3 hours prior to the infection. Infection was stopped after 6 hours and mRNA expression patterns were determined for genes labeled. Mean of samples are depicted with error bars of the standard deviation of each sample. Data are representative of at least three independent experiments.

IFN priming clearly enhances cDC activation suggesting that it may play an important role in the initiation of immunity. This function could likely be used to overcome viral immune inhibitors such as the NS1 protein from influenza virus that has been demonstrated to inhibit cytokine secretion and maturation in both mouse and human DCs. Therefore, we compared activation of cDCs by NS1 deficient PR8 virus (ΔNS1) to DCs infected with PR8 virus after a 3 hour pretreatment with 50–5,000 units/ml of IFN-β. Figure 7 demonstrates cDCs primed with IFN respond to wild type influenza virus at intensities comparable to an influenza virus lacking the IFN antagonist (ΔNS1). IFN priming can rescue the response to influenza for all cytokines and chemokines tested with the exception of IFN-β and TNF-α (Figure 7). These genes never reached the levels of ΔNS1 at any time point tested (0–12 hours) after 3-hour IFN-β pretreatment (data not shown). This may reflect differences in expression requirements for these proteins. Our data indicate that the priming effects of IFN-β counteract the inhibitory effects on DC activation genes induced by the influenza virus NS1 protein. Interestingly, IFN-β and TNF-α were still inhibited in the presence of the NS1 protein even when DCs were pretreated by IFN-β. While the transcription of both IFN-β and TNF-α is strongly dependent on NF-kβ activation other genes that are not so strongly dependent on this transcription factor can be induced by IFN-β treatment even in the presence of the influenza virus protein NS1 [19],[33].

## Discussion

Type I IFN has broad antiviral, immunological effects. It has been shown to impact NK and cytotoxic T cell elimination of virally infected cells, DC cross-presentation of viral antigens and B cell antibody production and isotype switching [34],[35]. Additionally, IFN-α/β has been found to alter pDC migration, development and maturation [36],[37],[38],[39]. However, the impact of IFN-β pretreatment on human DC activation by influenza virus infection had not been fully explored.

Osterlund and colleagues initially described an effect of IFN priming on DC responses to influenza virus [25]. In their studies they showed that pretreatment with IFN could enhance mRNA for type I and type III IFN and TNF [25]. These experiments were performed using high MOI of virus and did not show secreted protein data, leaving open the question of physiological relevance. In the current work, we have comprehensively examined the impact of type I IFN on the activation profiles of both subpopulations of DCs in the context of influenza virus infections and we demonstrate that IFN-β can potently enhance their response to virus induced activation in a dose and time dependent manner. Our data show that the priming effects of type I IFN on DCs impact both the levels of mRNA expression of IFN-responsive genes and the degree of viral replication. At all concentrations and time points explored, the low dose of IFN-β was able to impair viral replication but not able to completely eliminate this replication in DCs. This incomplete shut off may be necessary to allow DCs to be activated by the viral infection.

The novel question explored here was how DCs would respond to an influenza virus infection when they had been also exposed to type I IFN. In the context of a virus infection in vivo, it is very likely that epithelial cells may secrete type I IFN that can reach underlying DCs before the virus does. If the antiviral state had been initiated prior to or post infection, would DCs be activated by the viral infection or would the antiviral state block viral DC activation? Our results clearly demonstrate that both DC subsets are not only not impaired in their response to virus infection after exposure to type I IFN, but are primed by IFN-β, having increased activation following infection with an influenza virus.

The poor response of cDCs to wild type influenza virus infection in vitro is in contradiction to the immunological outcome of natural infection in vivo, since both humans and mice generate strong adaptive immunity and are able to clear influenza virus infection. Thus, DC activation must occur in vivo. Our data suggest that IFN priming may account for the ability of a host to respond to an infection that does not appear to elicit DCs activation in vitro. IFN priming could be a mechanism for the host to overcome the powerful ability of IFN antagonists such as the influenza NS1 protein to block IFN production, signaling and/or IFN-responsive genes actions. This has broad implications for the role of DC activation in the context of an antiviral immunological response. As shown by our data, very little viral replication is needed to elicit strong DC activation. This is in sharp contrast to cDC activation from viral infection in the absence of type I IFN, which is weak and viral dose dependent.

Our results suggest that pDCs also benefit from IFN signaling. Type I IFN has previously been shown to influence pDCs development [36], while in our studies we demonstrate that IFN has a substantial impact on the activation of pDCs following influenza virus infection. The importance of pDC activation, similar to the results of cDCs, is that despite very little viral input and replication, pDCs respond fully. This ability of pDCs to produce such large amounts of type I IFN with such small viral input, may be reflective of the role of pDCs during a natural infection. PDCs may be a host equivalent to IFN primed cDCs, in the sense that pDCs are not sensitive to the inhibitory effects of influenza virus IFN antagonist, the NS1 protein.

Despite the many similarities in IFN-β priming between the two subtypes of DCs, there were several important differences. cDCs demonstrated later priming kinetics with the majority of priming corresponding with viral replication. This delayed priming suggests several possible mechanisms. Priming may occur after 4 hours simply because input virus was not able to stimulate activation, and viral replication was necessary either to increase the amount of stimuli or to produce stimuli in a recognized structure. Another hypothesis for the late priming is that it occurs as a result of increased expression of IFN-responsive genes. One of the most likely proteins to account for cDC priming would be RIG-I, which is necessary for DC activation to influenza viruses [7]. The significance of crucial IFN-responsive genes acting as viral sensors, rather than other proteins involved in DC activation, like IRFs, is that IFN does not prime cDCs responses to TLR ligands (Table 1). This supports the notion that IFN priming in cDCs is augmenting the internal pathway of activation, most likely mediated by RIG-I. However, these two hypotheses of cDC priming are not mutually exclusive; and we propose that both may occur simultaneously. IFN priming in cDCs is dependent on viral replication being sensed by the RLR pathway and due to the increased expression of IFN-responsive genes like RIG-I, this internal pathway is able to stimulate a stronger cDC activation profile.

In contrast to the delayed cDC priming, pDCs demonstrate priming most substantially at 4 hours post infection and priming decreases with time. Again differing from cDCs, pDC activation did not follow the viral replication time course, suggesting a very different mechanism of priming than in cDCs. This finding is consistent with the profile of pDC activation by viruses being predominantly TLR dependent. In our experiments IFN-β priming in pDC was independent of viral replication and seen with both live virus and TLR ligand activation (Figures 3–6, and Table 1, bottom part). These results suggest that although IFN-β treatment did not enhance the TLR pathway in cDCs, IFN-β can enhance the overall activation within cells that utilize the TLR pathway as its primary viral sensor.

Lastly, the results from both pDCs and cDCs with IFN-β added post infection, demonstrate that while priming occurs over a broad time range, there is a point where the virus ‘wins’ and the enhancing effects of IFN-β treatment are not able to supplement the DC activation. It is possible that the viral sensors are made too late to be useful or they may not be made at all due to the inhibition of cellular machinery by the virus.

In summary, type I IFN priming overrides the inhibitory effects of viral antagonists on DC activation by eliciting strong responses in cDCs and even stronger responses in pDCs. The significance of this finding suggests the importance of evaluating DC responses in an environment similar to that in vivo. As DCs in vivo are responding to viruses in the context of setting that may contain multiple pro-inflammatory cytokines and chemokines, the effects of this environment cannot be disregarded. When evaluating a DC response, it is important to consider the actual stimuli the cells may have been exposed to prior to viral infection. Moreover, here we show that the establishment of antiviral state by type I IFN does not inhibit DC activation but rather, exerts priming effects, allowing for a more efficient detection and stronger response. Our data have important implications for the understanding of the initiation of immunity in the infected host, since differences in the micro-environment of the infected DC may account for different outcomes in adaptive immunity.

## Materials and Methods

### Viruses and cells

Influenza virus PR8 (H1N1) was grown in 9-day-old embryonated chicken eggs (SPAFAS; Charles River Laboratories). PR8 was titrated on MDCK cells by detection of hemagglutination (HA) activity in the supernatants after 48 h of infection, as previously described and by immunoflourescence, using a monoclonal antibody, PY102, specific for the HA protein (obtained from Jerome L. Schulman). All virus infections were performed in infection medium (Dulbecco's modified Eagle's medium, 0.35% bovine serum albumin, 0.12% NaHCO_3_, 100 µg/ml penicillin-streptomycin). For influenza virus titrations, 2.5 µg/ml trypsin was included in the infection medium.

MDCK and Vero cells were grown in tissue culture medium (Dulbecco's modified Eagle's medium [Invitrogen] with 10% fetal calf serum [HyClone], 1 mM sodium pyruvate [Invitrogen], 2 mM l-glutamine [Invitrogen], and 50 µg/ml gentamicin [Invitrogen]). All cells were grown at 37°C in 7% CO_2_.

### Isolation and culture of human DCs

Peripheral blood mononuclear cells were isolated by Ficoll density gradient centrifugation (Histopaque; Sigma Aldrich) from buffy coats of healthy human donors (Mount Sinai Blood Donor Center and New York Blood Center). CD14+ cells were immunomagnetically purified using anti-human CD14 antibody-labeled magnetic beads and BDCA4+ cells were immunomagnetically purified using anti-human BDCA4 (CD304)^+^ antibody- labeled magnetic beads and iron-based Midimacs LS columns (Miltenyi Biotec). After elution from the columns, CD14+ cells were plated (0.7×10^6^ cells/ml) in DC medium (RPMI [Invitrogen], 10% fetal calf serum [HyClone] or 4% human serum [Cambrex], 100 units/ml of penicillin, and 100 µg/ml streptomycin [Invitrogen]) supplemented with 500 U/ml human granulocyte-macrophage colony-stimulating factor (GM-CSF; Peprotech) and 1,000 U/ml human interleukin-4 (IL-4; Peprotech) and incubated for 5 to 6 days at 37°C. Our cultured DCs were routinely 95–98% positive for CD11c as tested by flow cytometry, from over 40 independent isolations. BDCA4^+^ cells were treated immediately following elution. PDCs were tested for purity by flow cytometry. Briefly, BDCA4^+^ cells were stained with fluorescein isothiocyanate FITC)-linked CD123 and phycoerythrin (PE)-linked BDCA2 (CD303), according to the manufacturer's instructions (Miltenyi Biotec), and the expression of each marker was determined by flow cytometry using an FC500 flow cytometer from Beckman Coulter. Data were analyzed using Flowjo software. The average purity of BDCA4+ cells was 91.07±5.01% as defined as double positive for CD123 and BDCA2 (CD303) with n = 63. Each experiment used an independent donor with no overlap between pDC and cDC donors.

### Infection and treatment of DCs

Immediately following isolation for BDCA4+ cells and after 5 to 6 days in culture for the CD14+ cells, DCs were either pre-treated with 5 to 5,000 U/ml IFN- β (PBL) and/or were treated with one of the following: live influenza PR8 virus at a multiplicity of infection (MOI) of 0.5, UV-inactivated influenza virus at a MOI = 5, 500 ng/ml LPS (Sigma-Aldrich), 6 ug/ml CpG (Coley Pharmaceutical Group), 2.5 ug/ml poly (I∶C) (InvivoGen), 0.5 ug/ml CL-075 (InvivoGen), 1 ug/ml Gardiquimod (InvivoGen). Cells were treated in medium (RPMI [Invitrogen], 4% human serum [Cambrex], 100 units/ml of penicillin, and 100 µg/ml streptomycin [Invitrogen]) at 1×10^6^ cells/ml for different time periods, depending on the experiment. In experiments in which the IFN media was removed, fresh media was added prior to viral infection.

### Capture ELISAs

Capture enzyme-linked immunosorbent assays (ELISAs) for IFN-α, TNF-α, IL-6, IL-8, RANTES, IP-10 and MIP1-β (Upstate/Millipore) were performed as part of a multiplex assay following the manufacturer's protocol. Plates were read in a Luminex plate reader, and data were analyzed using software from Applied Cytometry Systems. All samples were assayed in duplicate or triplicate.

### RNA extraction from human DCs

Samples of 0.15×10^6^ to 0.5×10^6^ DCs differentially treated according to the experimental protocol were pelleted, and RNA was isolated and treated with DNase by using an Absolutely RNA RT-PCR micro prep kit (Stratagene). RNA was quantified using a Nanodrop spectrophotometer (Nanodrop Technologies).

### Quantitative real-time PCR

qRT-PCR of the extracted RNAs was performed by using a previously published SYBR green protocol with an ABI7900 HT thermal cycler by the Mount Sinai Quantitative PCR Shared Research Facility. Each transcript in each sample was assayed in triplicate, and the mean cycle threshold was used to calculate the *x*-fold change and control changes for each gene. Three housekeeping genes were used for global normalization in each experiment (actin, Rps11, and tubulin genes). Data validity by modeling of reaction efficiencies and analysis of measurement precision was determined as described previously [17].

### Statistical Analyses

Statistical analyses were performed using student's two-tailed *t* test. Unless otherwise indicated, means±standard deviation for each sample are shown.

## Supporting Information

Figure S1 IFN-β priming seen in pDCs response to TLR7 ligand. pDCs were pretreated with IFN-β (50 units/ml) for 3 hours. Following pretreatment, the IFN media was removed and cells were treated with Gardiquimod (IFN+Gard) for 3 hours. Control pDCs were either treated with Gardiquimod only (Gard), pretreated with IFN only (IFN), or neither (NI). (A) Copy number of mRNA expression values are depicted for the specific gene labeled. (B) Protein secretion amounts from multiplex ELISAs. Mean of samples are depicted with error bars of the standard deviation of each sample. Data is representative of at least three independent experiments. All samples have student t test p<0.05 between the IFN+PR8 condition and the other conditions, with the exception of IL-6 mRNA expression.
(0.79 MB TIF)

## References

[1] Banchereau J, Briere F, Caux C, Davoust J, Lebecque S (2000). Immunobiology of dendritic cells.. Annu Rev Immunol.

[2] Banchereau J, Steinman RM (1998). Dendritic cells and the control of immunity.. Nature.

[3] Cella M, Sallusto F, Lanzavecchia A (1997). Origin, maturation and antigen presenting function of dendritic cells.. Curr Opin Immunol.

[4] Kawai T, Akira S (2006). Innate immune recognition of viral infection.. Nat Immunol.

[5] Akira S, Takeda K (2004). Toll-like receptor signalling.. Nat Rev Immunol.

[6] Lee MS, Kim YJ (2007). Pattern-recognition receptor signaling initiated from extracellular, membrane, and cytoplasmic space.. Mol Cells.

[7] Kato H, Sato S, Yoneyama M, Yamamoto M, Uematsu S (2005). Cell type-specific involvement of RIG-I in antiviral response.. Immunity.

[8] Lopez CB, Moltedo B, Alexopoulou L, Bonifaz L, Flavell RA (2004). TLR-independent induction of dendritic cell maturation and adaptive immunity by negative-strand RNA viruses.. J Immunol.

[9] Colonna M, Trinchieri G, Liu YJ (2004). Plasmacytoid dendritic cells in immunity.. Nat Immunol.

[10] Cao W, Liu YJ (2007). Innate immune functions of plasmacytoid dendritic cells.. Curr Opin Immunol.

[11] Hardy MP, Owczarek CM, Trajanovska S, Liu X, Kola I (2001). The soluble murine type I interferon receptor Ifnar-2 is present in serum, is independently regulated, and has both agonistic and antagonistic properties.. Blood.

[12] Tough DF (2004). Type I interferon as a link between innate and adaptive immunity through dendritic cell stimulation.. Leuk Lymphoma.

[13] Der SD, Zhou A, Williams BR, Silverman RH (1998). Identification of genes differentially regulated by interferon alpha, beta, or gamma using oligonucleotide arrays.. Proc Natl Acad Sci U S A.

[14] Kang DC, Gopalkrishnan RV, Wu Q, Jankowsky E, Pyle AM (2002). mda-5: An interferon-inducible putative RNA helicase with double-stranded RNA-dependent ATPase activity and melanoma growth-suppressive properties.. Proc Natl Acad Sci U S A.

[15] Basler CF, Garcia-Sastre A (2002). Viruses and the type I interferon antiviral system: induction and evasion.. Int Rev Immunol.

[16] Garcia-Sastre A, Egorov A, Matassov D, Brandt S, Levy DE (1998). Influenza A virus lacking the NS1 gene replicates in interferon-deficient systems.. Virology.

[17] Fernandez-Sesma A, Marukian S, Ebersole BJ, Kaminski D, Park MS (2006). Influenza virus evades innate and adaptive immunity via the NS1 protein.. J Virol.

[18] Kochs G, Garcia-Sastre A, Martinez-Sobrido L (2007). Multiple anti-interferon actions of the influenza A virus NS1 protein.. J Virol.

[19] Wang X, Li M, Zheng H, Muster T, Palese P (2000). Influenza A virus NS1 protein prevents activation of NF-kappaB and induction of alpha/beta interferon.. J Virol.

[20] Lopez CB, Garcia-Sastre A, Williams BR, Moran TM (2003). Type I interferon induction pathway, but not released interferon, participates in the maturation of dendritic cells induced by negative-strand RNA viruses.. J Infect Dis.

[21] Brimnes MK, Bonifaz L, Steinman RM, Moran TM (2003). Influenza virus-induced dendritic cell maturation is associated with the induction of strong T cell immunity to a coadministered, normally nonimmunogenic protein.. J Exp Med.

[22] Montoya M, Edwards MJ, Reid DM, Borrow P (2005). Rapid activation of spleen dendritic cell subsets following lymphocytic choriomeningitis virus infection of mice: analysis of the involvement of type 1 IFN.. J Immunol.

[23] Pollara G, Jones M, Handley ME, Rajpopat M, Kwan A (2004). Herpes simplex virus type-1-induced activation of myeloid dendritic cells: the roles of virus cell interaction and paracrine type I IFN secretion.. J Immunol.

[24] Montoya M, Schiavoni G, Mattei F, Gresser I, Belardelli F (2002). Type I interferons produced by dendritic cells promote their phenotypic and functional activation.. Blood.

[25] Osterlund P, Veckman V, Siren J, Klucher KM, Hiscott J (2005). Gene expression and antiviral activity of alpha/beta interferons and interleukin-29 in virus-infected human myeloid dendritic cells.. J Virol.

[26] Yount JS, Moran TM, Lopez CB (2007). Cytokine-independent upregulation of MDA5 in viral infection.. J Virol.

[27] Honda K, Sakaguchi S, Nakajima C, Watanabe A, Yanai H (2003). Selective contribution of IFN-alpha/beta signaling to the maturation of dendritic cells induced by double-stranded RNA or viral infection.. Proc Natl Acad Sci U S A.

[28] Dalod M, Hamilton T, Salomon R, Salazar-Mather TP, Henry SC (2003). Dendritic cell responses to early murine cytomegalovirus infection: subset functional specialization and differential regulation by interferon alpha/beta.. J Exp Med.

[29] Theofilopoulos AN, Baccala R, Beutler B, Kono DH (2005). Type I interferons (alpha/beta) in immunity and autoimmunity.. Annu Rev Immunol.

[30] Gresser I, Tovey MG, Maury C, Bandu MT (1976). Role of interferon in the pathogenesis of virus diseases in mice as demonstrated by the use of anti-interferon serum. II. Studies with herpes simplex, Moloney sarcoma, vesicular stomatitis, Newcastle disease, and influenza viruses.. J Exp Med.

[31] Yount JS, Kraus TA, Horvath CM, Moran TM, Lopez CB (2006). A novel role for viral-defective interfering particles in enhancing dendritic cell maturation.. J Immunol.

[32] Lopez CB, Fernandez-Sesma A, Czelusniak SM, Schulman JL, Moran TM (2000). A mouse model for immunization with ex vivo virus-infected dendritic cells.. Cell Immunol.

[33] Zheng Y, Ouaaz F, Bruzzo P, Singh V, Gerondakis S (2001). NF-kappa B RelA (p65) is essential for TNF-alpha-induced fas expression but dispensable for both TCR-induced expression and activation-induced cell death.. J Immunol.

[34] Le Bon A, Tough DF (2002). Links between innate and adaptive immunity via type I interferon.. Curr Opin Immunol.

[35] Stetson DB, Medzhitov R (2006). Type I interferons in host defense.. Immunity.

[36] Asselin-Paturel C, Brizard G, Chemin K, Boonstra A, O'Garra A (2005). Type I interferon dependence of plasmacytoid dendritic cell activation and migration.. J Exp Med.

[37] Zuniga EI, McGavern DB, Pruneda-Paz JL, Teng C, Oldstone MB (2004). Bone marrow plasmacytoid dendritic cells can differentiate into myeloid dendritic cells upon virus infection.. Nat Immunol.

[38] Toma-Hirano M, Namiki S, Miyatake S, Arai KI, Kamogawa-Schifter Y (2007). Type I interferon regulates pDC maturation and Ly49Q expression.. Eur J Immunol.

[39] Dalod M, Salazar-Mather TP, Malmgaard L, Lewis C, Asselin-Paturel C (2002). Interferon alpha/beta and interleukin 12 responses to viral infections: pathways regulating dendritic cell cytokine expression in vivo.. J Exp Med.

