# Supplementary figures and images for: Interferon-β Pretreatment of Conventional and Plasmacytoid Human Dendritic Cells Enhances Their Activation by Influenza Virus
Source: PLoS Pathog. 2008 Oct 31;4(10):e1000193. doi: 10.1371/journal.ppat.1000193 (PMC2568957; doi:10.1371/journal.ppat.1000193)

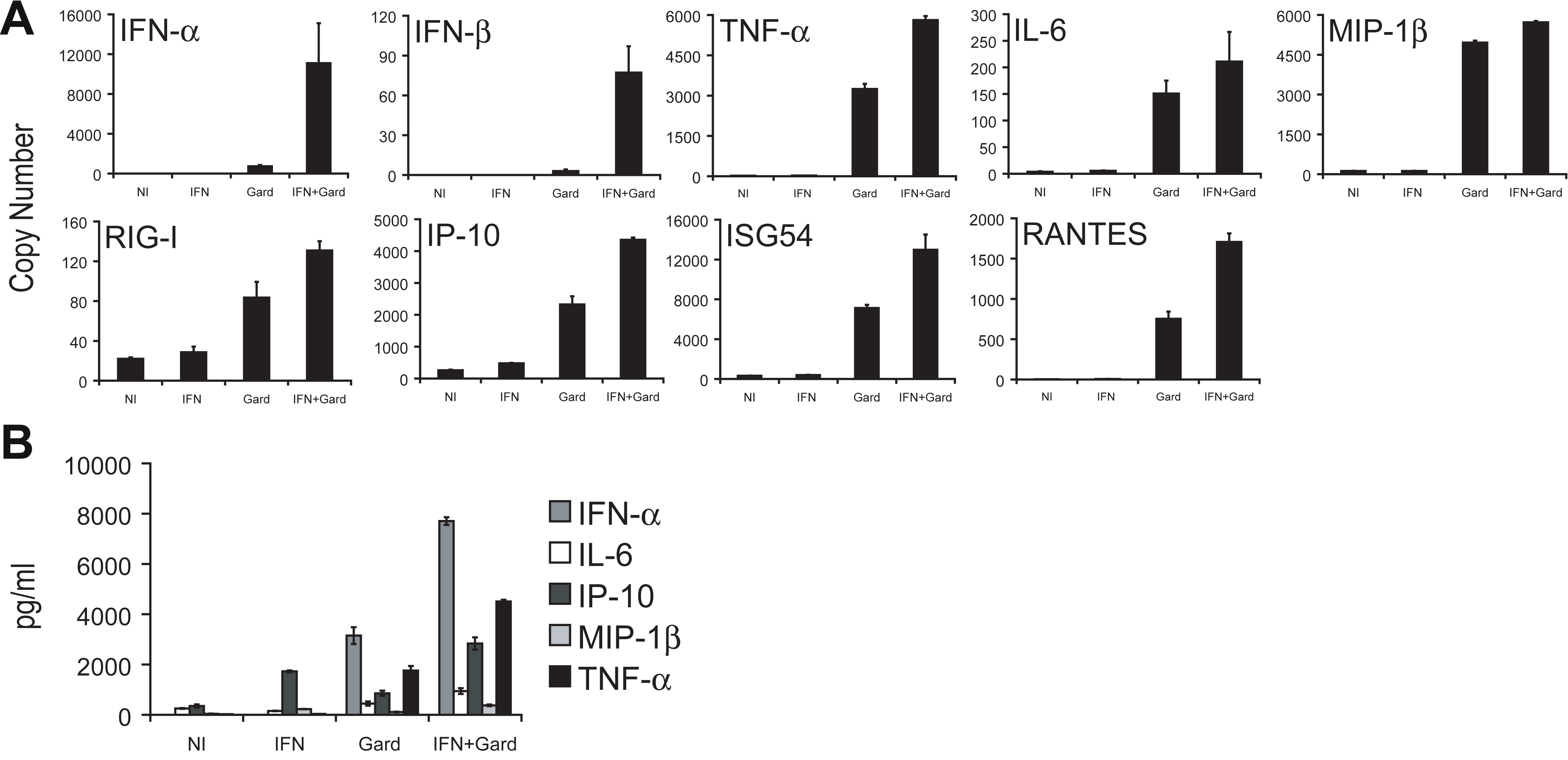

Supplement: Figure S1 — IFN-β priming seen in pDCs response to TLR7 ligand. pDCs were pretreated with IFN-β (50 units/ml) for 3 hours. Following pretreatment, the IFN media was removed and cells were treated with Gardiquimod (IFN+Gard) for 3 hours. Control pDCs were either treated with Gardiquimod only (Gard), pretreated with IFN only (IFN), or neither (NI). (A) Copy number of mRNA expression values are depicted for the specific gene labeled. (B) Protein secretion amounts from multiplex ELISAs. Mean of samples are depicted with error bars of the standard deviation of each sample. Data is representative of at least three independent experiments. All samples have student t test p<0.05 between the IFN+PR8 condition and the other conditions, with the exception of IL-6 mRNA expression. (0.79 MB TIF) [file ppat.1000193.s001.tif]
